# Effectiveness of Push–Pull Systems to Fall Armyworm (*Spodoptera frugiperda*) Management in Maize Crops in Morelos, Mexico

**DOI:** 10.3390/insects12040298

**Published:** 2021-03-29

**Authors:** Ouorou Ganni Mariel Guera, Federico Castrejón-Ayala, Norma Robledo, Alfredo Jiménez-Pérez, Georgina Sánchez-Rivera, Lilia Salazar-Marcial, Hilda Elizabet Flores Moctezuma

**Affiliations:** Laboratorio de Ecología Química de Insectos, Centro de Desarrollo de Productos Bióticos, Instituto Politécnico Nacional, Calle CeProBi No. 8, San Isidro, 62739 Yautepec, Mexico; aljimenez@ipn.mx (A.J.-P.); gsanchezri@ipn.mx (G.S.-R.); lsalazarm@ipn.mx (L.S.-M.); hfloresm@ipn.mx (H.E.F.M.)

**Keywords:** agroecology, attractive plants, repellent plants, sustainability

## Abstract

**Simple Summary:**

Fall Armyworm (*Spodoptera frugiperda*) is an insect with generalist habits that causes serious damage to important agricultural crops, among which is maize (*Zea mays*). Given the obvious consequences of conventional agriculture and the limitations of organic agriculture, agroecological management strategies such as Push–Pull are increasingly considered since, in addition to production purposes, these systems channel economic, ecological, and social viability of that production. The successful introduction of these systems, still little implemented outside Africa, necessarily requires field effectiveness studies of laboratory and/or greenhouse proposals. Therefore, this study evaluated the field effectiveness of Push–Pull strategies designed for the management of *S*. *frugiperda* in maize crops in Morelos, Mexico. Most of the evaluated systems presented lower levels of fall armyworm infestations than those of the maize monoculture. *Mombasa*—*D*. *ambrosioides*, *Mulato II*—*T*. *erecta*, *Mulato II*—*C*. *juncea*, *Tanzania*—*T*. *erecta* and *Tanzania*—*D*. *ambrosioides* systems presented higher yields and profits than those observed in monocultures.

**Abstract:**

Chemical control is the main method used to combat fall armyworm in maize crops. However, its indiscriminate use usually leads to a more complex scenario characterized by loss of its effectiveness due to the development of resistance of the insect pest, emergence of secondary pests, and reduction of the populations of natural enemies. For this reason, efforts to develop strategies for agroecological pest management such as Push–Pull are increasingly growing. In this context, the present study was carried out to evaluate field effectiveness of Push–Pull systems for *S*. *frugiperda* management in maize crops in Morelos, Mexico. In a randomized block experiment, the incidence and severity of *S*. *frugiperda*, the development and yield of maize were evaluated in nine Push–Pull systems and a maize monoculture. The Push–Pull systems presented incidence/severity values lower than those of the monoculture. Morphological development and maize yield in the latter were lower than those of most Push–Pull systems. *Mombasa*—*D*. *ambrosioides*, *Mulato II*—*T*. *erecta*, *Mulato II*—*C*. *juncea*, *Tanzania*—*T*. *erecta* and *Tanzania*—*D*. *ambrosioides* systems presented higher yields than those of monocultures.

## 1. Introduction

Agroecological production systems are biodiverse, resilient, energy efficient, socially just and constitute a basis for food security and sovereignty [1,2]. In Latin America, including Mexico, a polyculture traditionally made up of maize (*Zea mays* L.), beans (*Phaseolus vulgaris* L.) and squash (*Cucurbita* spp.) that meets these characteristics is the Milpa [3]. This system, in addition to promoting agroecological and cultural diversity [4], is resistant to pests and diseases [5]. However, as part of agriculture sedentarization, the traditional milpa (of a migratory nature) is being replaced, even among small farmers and indigenous peoples, by a modern semi-permanent agriculture. In this context, it is necessary to implement semi-permanent agricultural systems that maintain the Milpa system benefits and are resistant to *Spodoptera frugiperda*, the major pest of maize in Mexico.

Among the agroecological production systems most adopted today, Push–Pull systems stand out [6,7,8,9]. The term Push–Pull arises from the pioneering studies of Pyke et al. [10] and Miller and Cowles [11]. The proposal made by these studies, despite their effectiveness, was not widely implemented due to various factors, including the high tolerance to the use of agrochemicals at that time. The most effective and therefore most widely implemented Push–Pull method is that developed by Khan et al. [12] in Kenya to combat *Chilo partellus* and *Striga hermonthica* in maize crops. This system, in addition to controlling the pest (*C*. *partellus*) and weeds (*S*. *hermonthica*), improves the soils, allows the production of other resources (forage) and doubles maize yield. The consideration of these additional goals took the Push–Pull to another dimension, making it an attractive agroecological management method and not just one of pest control.

Although this strategy is being widely adopted in many African countries, its implementation is still scarce outside the limits of that continent. The success of the Push–Pull system depends on the components (attractants and repellents) of the system that vary depending on the pathosystem, environmental factors, and the biotic and abiotic resources available in a certain region. For this reason, for the *Zea mays*—*Spodoptera frugiperda* pathosystem, in which Push–Pull has been shown to be efficient in Africa [13,14,15], studies were recently carried out with local and/or naturalized species [16]. The results of this study indicate that *Brachiaria hybrid* cv *Mulato II*, *Panicum maximum* cv *Mombasa*, *Panicum maximum* cv *Tanzania* could be suitable trap plants and *Dysphania ambrosioides*, *Tagetes erecta* and *Crotalaria juncea* suitable intercrops in Push–Pull systems for *S*. *frugiperda* management in maize crops in Morelos, Mexico. However, the field effectiveness of these has not been evaluated.

Based on the above, the present study was carried out to evaluate the field effectiveness of these local Push–Pull variants for the management of *S*. *frugiperda* in maize crops in Yaupetec, Morelos, Mexico.

## 2. Materials and Methods

### 2.1. Study Area Characterization

**Figure 1:**
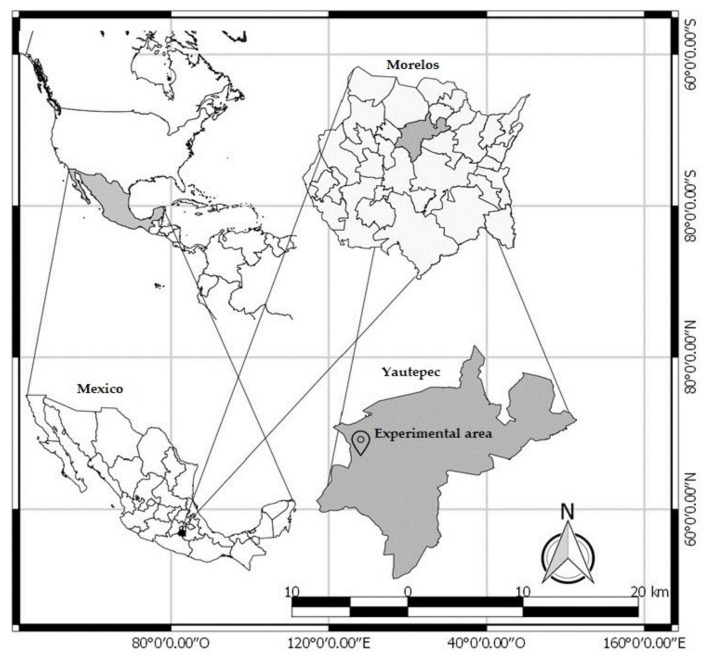
Location of the study area, Municipality of Yautepec, Morelos, Mexico.

**Figure 2:**
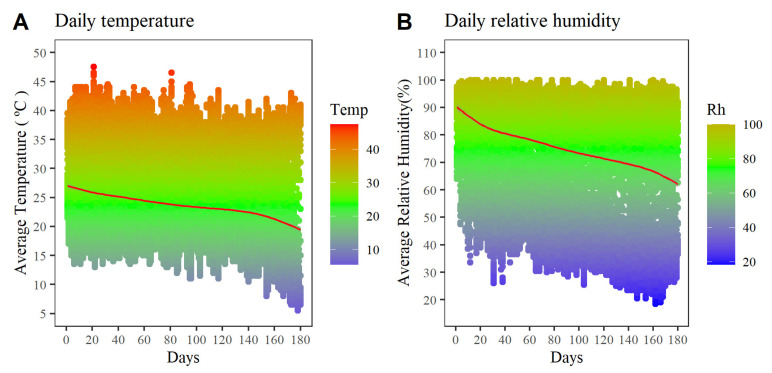
Evolution of temperature and relative humidity during the experiment at Yautepec Municipality, Morelos, Mexico.

The present study was carried out in an area of 6341 m^2^ located in maize production areas of the Lomas del Potrero neighborhood, Yautepec municipality, Morelos state, Mexico, with geographic coordinates between 18.893323 N and 99.102158 W (Figure 1). Yautepec has a tropical savanna climate (Aw), according to the Köppen-Geiger classification, an annual rainfall of 928 mm and an average temperature of 23.2 °C [17]. The temperature and relative humidity of the area (Figure 2) were monitored throughout the experiment (20 June 2019–16 December 2019) with a Lascar EL-USB-2 datalogger (Accuracy: ±0.45 °C/±2.05% RH) programmed for a recording frequency of 10 min. During the experimental period, the area presented an average temperature of 23.69 ± 1.55 °C and average relative humidity of 74.90 ± 4.94%.

### 2.2. Experimental Design

**Figure 3:**
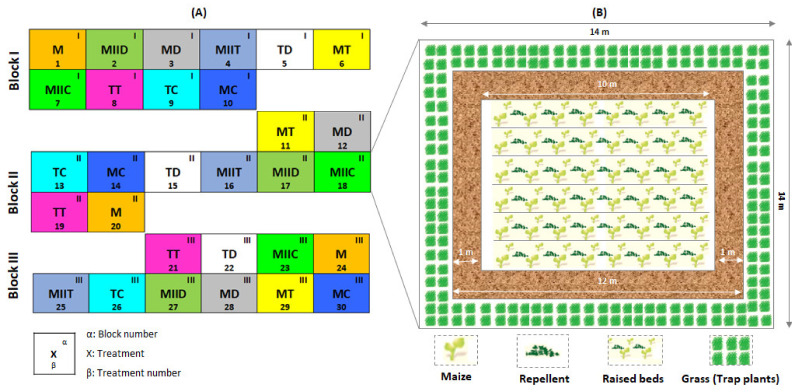
Experimental units (**B**) established in a randomized block experiment (**A**). For abbreviations, see Table 1.

Ten treatments (Table 1) were evaluated in experimental units (14 m × 14 m plots) made up of 8 beds (1.06 m wide and 10 m long) established in a randomized complete block design (Figure 3A). This design accounted for the fertility gradient observed in the experimental area and the size of the plots was chosen to comply with the minimum size of 10 m × 10 m recommended by Khan [18] for Push–Pull plots.

In each of the experimental units equidistant of 2 m in each block, the maize planting area (10 m × 10 m) was made up of 6 beds of maize (Pioneer P3966W) as the main crop and an intercrop (repellent). At one meter from the maize planting area of each plot, a grass border (Fall armyworm trap plant) was established (Figure 3B). The plots establishment data are summarized in the Table S1. All treatments presented weed infestation, which was controlled manually every fifteen days until canopy closure in each plot.

### 2.3. Incidence and Severity of Spodoptera Frugiperda Damage

In each plot, samplings were carried out at 7, 14 and 21 days after maize germination (DAG) with the five-point sampling method, checking 10 plants at each point (Figure S1). The number of damaged plants was used to determine the incidence (Equation (1)—[19]). Severity was evaluated by visual observation of the degree of leaf damage. For this, a severity scale made up of five categories was used [13,15]: 1. damage-free plants (plants without visual symptoms of damage); 2. plants with low damage (plants with leaf area damage less than 25%); 3. plants with medium damage (plants with leaf area damage between 25% and 50%); 4. plants with high damage (plants with leaf area damage between 50% and 75%); and 5. plants with very high or severe damage (plants with more than 75% of their leaf area damaged). Unlike the incidence that was evaluated once a week during the crop’s first three weeks, the severity (Equation (2)—[19]) was only evaluated in the third week after maize germination (at 21 days).

(1) $$I\;\left(\%\right)=\frac{NDP}{NPE}*100$$

(2) $$S\left(\%\right)=\frac{\sum NPDD*DD}{NPE*GDD}*100$$

where: *I* = Incidence; *NDP* = Number of damaged plants; *NPE* = number of plants evaluated; *S* = severity; *NPDD* = number of plants damaged to a certain degree of damage; *DD* = degree of damage; *GDD* = greater degree of damage.

The percent reduction of Fall Armyworm damage by Push–Pull systems over the control (monoculture) was determined by Equation (3) [20].

(3) $$PR=\frac{\left(C-PPS\right)}{C}\times 100$$

where: PR is the percent reduction of Fall Armyworm incidence (FAIR) or severity (FASR) over monoculture; C is Fall Armyworm incidence or severity in the control (monoculture) and PPS is Fall Armyworm incidence or severity in one of the Push–Pull systems. 

### 2.4. Morphoagronomic and Edaphoclimatic Variables in the Production Systems

Mixed cropping can affect maize crops’ growth. Therefore, after the vegetative phase, approximately three months after the establishment of the crops, stem diameter (Dt), total height (Ht), cob diameter (De) and cob length (Le) were measured in ten plants selected at random in each treatment. Leaf Area Index—Equation (4) [21] was determined in each of the systems using the Beer–Lambert absorption law [22]. Measurements of transmitted and incident photosynthetically active radiation (RFAt y RFAi) were made with a digital meter (^®^Steren) at 65 days of cultivation, in the center of each plot, between 12:00 p.m. and 2:00 p.m.

(4) $$LAI=-\frac{Ln\left(\frac{I}{I_{0}}\right)}{k}$$

where: *LAI* = Leaf Area Index; *k* = light extinction coefficient ~0.60; *I* = transmitted photosynthetically active radiation (over the canopy); *I*_0_ = incident photosynthetically active radiation (below canopy).

The sustainability of an agroecosystem depends on efficient water management [2]. Therefore, soil moisture and temperature were measured after canopy closure in the plots, after a canicular period of approximately 2 weeks. Soil moisture was measured with a Kelway meter (Accuracy: ±10%) and soil temperature with a thermometer ^®^Taylor (depth: 13 cm; Accuracy: ±2°). Five measurements of these variables were made in each plot, following the five-point sampling method (Figure S1).

### 2.5. Maize Grains and Green Forage Yields in the Systems

Maize grain yield was determined by manually harvesting two beds in each plot [23]. The cobs were shelled with a mechanical sheller and weighed with a hanging digital scale. At 153 and 168 days after maize germination, the moisture content of the grains was determined with an electric meter (^®^ AGROFARM DK-6064). The harvest was carried out in the sixth month (December 16) with approximately a moisture content of 15% and the yields were adjusted to 14%. The main dates of establishment of the experiment and maize harvest are shown in Table S1.

The yield of *C*. *juncea* was determined by harvesting the production of the central bed (1 m × 10 m) from each of the 9 plots that make up this species. Regarding the pastures (*Mombasa*, *Tanzania*, *Mulato II*), their yield was determined by the quadrant method, which consisted of cutting and weighing all the grass within a 1 m^2^ quadrat [24]. A sample was taken in each of the 9 plots of each grass species.

### 2.6. Comparative Analysis of Profitability of the Production Systems

The cost generated using a certain pest control technique must be less than the damage that the pest would cause if nothing were done [25]. Therefore, at harvest of maize, the profitability of each system (maize crop + Push–Pull strategy) was evaluated by the Net Present Value (NPV, Equation (5)) and the Benefit/Cost Ratio (BCR, Equation (6)), both estimated at a discount rate of 9.5% [26]. The costs and income considered for the determination of these criteria are summarized in Table S2.

(5) $$NPV=\overset{n}{\underset{t=0}{\sum }}\frac{B_{t}}{{\left(1+r\right)}^{n}}-\overset{n}{\underset{t=0}{\sum }}\frac{C_{t}}{{\left(1+r\right)}^{n}}$$

(6) $$BCR=\overset{n}{\underset{t=0}{\sum }}\frac{B_{t}}{{\left(1+r\right)}^{n}}/\overset{n}{\underset{t=0}{\sum }}\frac{C_{t}}{{\left(1+r\right)}^{n}}$$

where: $C_{t}=$ Cost at the period t; $B_{t}=$ Benefit at the period t; r= Discount Rate; n = number of periods; t= *t*th period (months).

### 2.7. Statistical Analysis

Data analyses were preceded by verification of compliance with the assumptions of normality and homoscedasticity for all the variables studied. These were verified with the Shapiro–Wilk [27] and Levene [28] tests, respectively. The analyses were carried out using mixed models, considering the treatments as fixed effects and the nested samples taken within each experimental plot as random effects. The analysis of variance of the variables that fulfilled the assumption of normality (Dt, Ht, De, Le, LAI, soil temperature, soil moisture, maize grain yield and green forage yield) were carried out using Linear Mixed Model—LMM (Equation (7), [29]). The variables (incidence and severity) that did not meet this assumption were analyzed with a Generalized Linear Mixed Model—GLMM (Equation (8), [29]), using the Logit link function and the Beta error distribution model, as recommended by Chen et al. [30] for data in proportions or percentages. The LMM and GLMM models were fitted, respectively, with the *lmer ()* and *glmer ()* functions of the *lme4* package [31]. Pairwise comparisons of means were carried out with the Scott–Knott [32] test performed using the *ScottKnott* package [33]. This test, in addition to being robust to non-compliance with normality assumption [34], is appropriate when there is interest in separating groups of means, without ambiguity in the results, in experiments with a large number of treatments [35].

(7) $$E\left[Y_{j}|u_{i},\ldots ,\;u_{q}\right]=\beta_{0}+\overset{p}{\underset{i=1}{\sum }}\beta_{i}x_{ij}+\overset{q}{\underset{k=1}{\sum }}z_{kj}u_{k},\;j=1,\ldots ,n$$

where: $Y_{j}$ is a response variable with normal distribution, $\beta_{i}$ is the *i*th fixed-effect coefficient, $x_{ij}$ is the *i*th fixed-effect explanatory variable for the *j*th observation, and $z_{kj}$ is the binary indicator variable for the effect of the *k*th random effect, $u_{k}$, on the *j*th observation.

(8) $$\eta_{ij}=g\left(E\left[Y_{j}|u_{i},\ldots ,\;u_{q}\right]\right)=\beta_{0}+\overset{p}{\underset{i=1}{\sum }}\beta_{i}x_{ij}+\overset{q}{\underset{k=1}{\sum }}z_{kj}u_{k},\;j=1,\ldots ,n$$

where: $Y_{j}$ is a response variable whose conditional distribution given the random effects belongs to the exponential family or can be written as a quasi-likelihood, where $\beta_{0}$ is the overall mean, $\beta_{i}$ is the ith fixed-effect coefficient,$\;x_{ij}$ is the *i*th fixed-effect explanatory variable on the *j*th observation, $z_{kj}$ is the binary indicator variable for the effect of the *k*th random effect, $u_{k}$, on the *j*th observation, and *g*(*⋅*) is the link function relating the conditional mean of the response to the predictors.

The differences between the management systems were observed in the biplot of a principal component analysis (PCA) based on the variables studied in the systems. The PCA was performed with the function *prcomp ()* of the package *stats* and the biplot was constructed with the function *fviz_pca_biplot ()* of the package *FactoMineR* [36]. To calculate the profitability criteria, the *FinCal* package [37] was used. All statistical analyses were performed in R 4.0.2 [38]. 

## 3. Results

### 3.1. Incidence and Severity of Fall Armyworm in the Evaluated Systems

**Figure 4:**
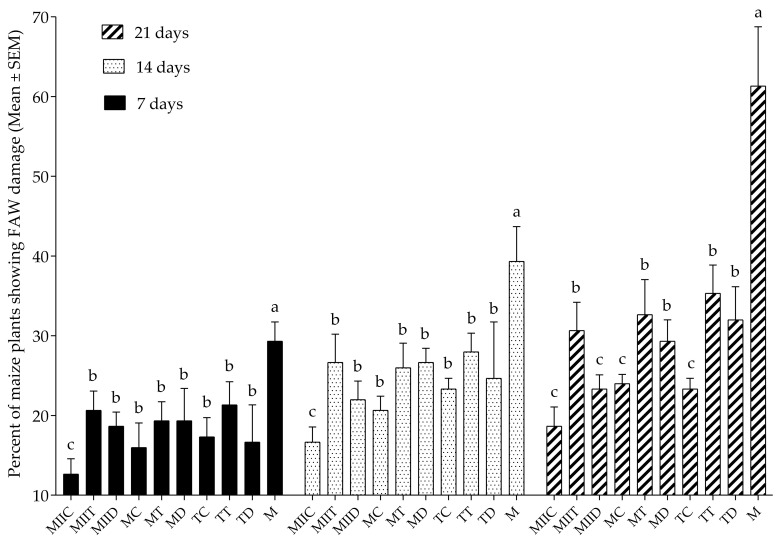
Incidence of *S*. *frugiperda* in maize crops in the systems evaluated at 7, 14 and 21 days. Different letters indicate a significant difference by the Scott–Knott test (α = 0.05). For abbreviations, see Table 1.

Significant differences were observed between *S*. *frugiperda* (Fall Armyworm (FAW)) incidence in the systems at 7, 14 and 21 days (Figure 4). During the three dates, the lowest incidences were recorded in the MIIC system and the highest in the monoculture.

**Figure 5:**
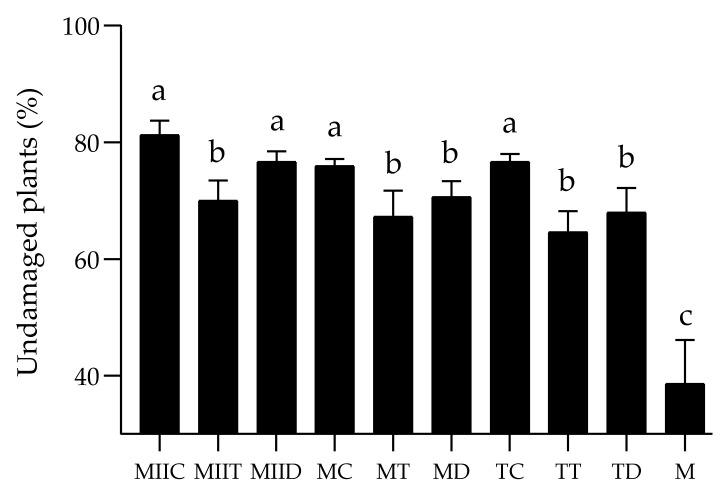
Percentage of undamaged plants in the different treatments. Different letters indicate a significant difference by the Scott–Knott test (α = 0.05). For abbreviations, see Table 1.

**Figure 6:**
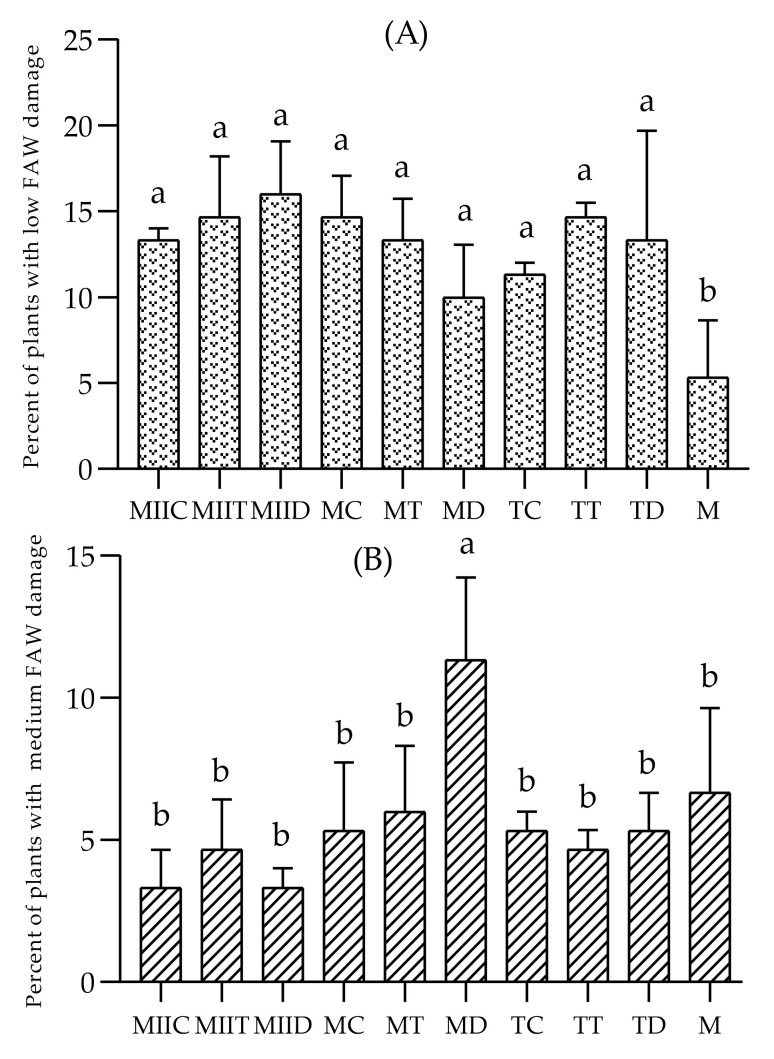

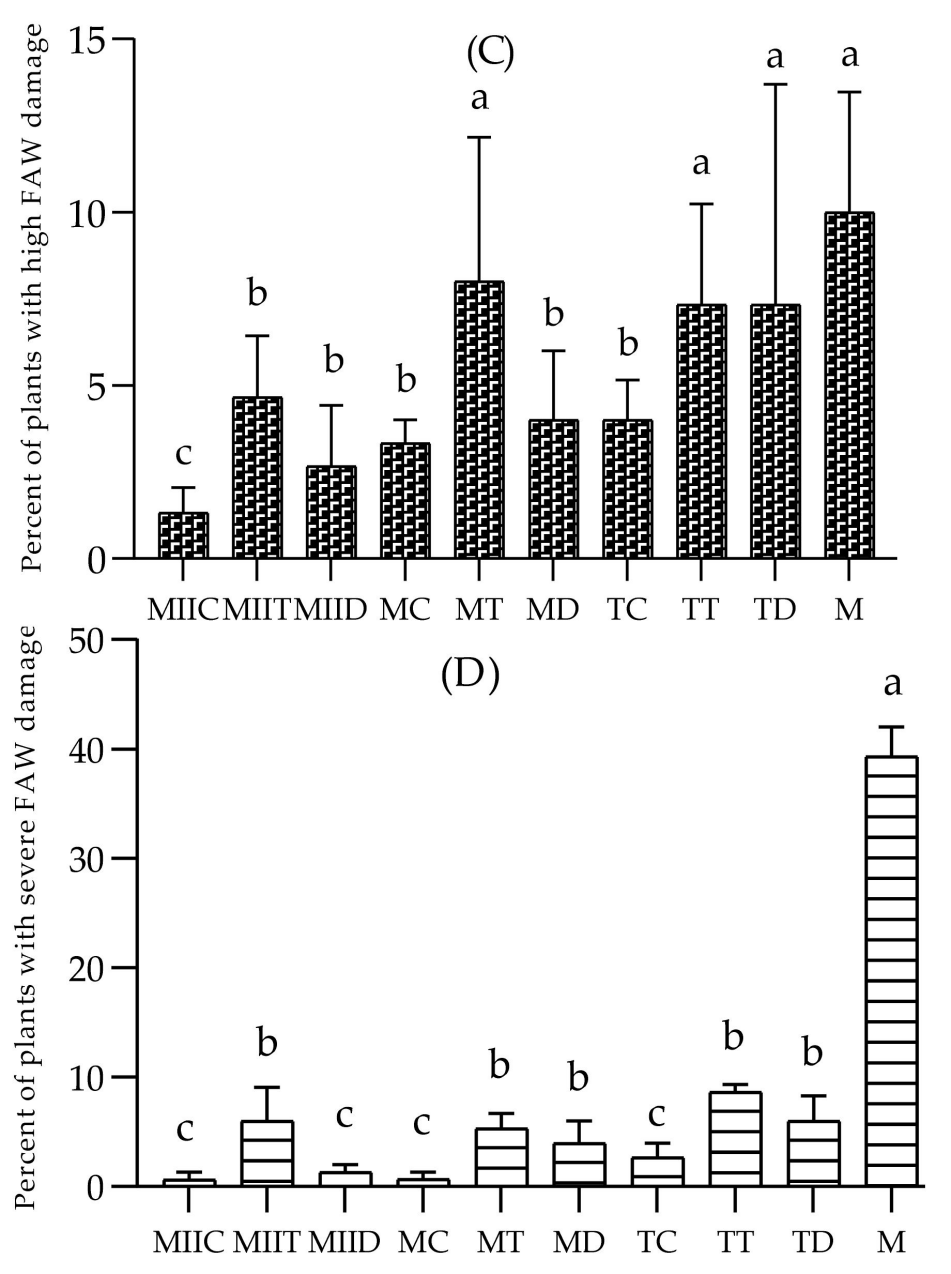
Severity of *S*. *frugiperda* in maize crops in the evaluated systems. Percent of plants with Fall Armyworm Low damage (**A**), Medium damage (**B**), High damage (**C**) and Severe damage (**D**). Different letters indicate a significant difference by the Scott–Knott test (α = 0.05). For abbreviations, see Table 1.

This same trend is observed in Figure 5, which indicates that monoculture presented the lowest proportion of undamaged plants, and MIIC, MIID, TC and MC systems, the highest proportions. The monoculture presented the highest proportion of plants with high and severe damage (Figure 6C,D). The proportions of plants with low damage in the Push–Pull systems were significantly higher than those recorded in the maize monoculture (Figure 6A).

### 3.2. Morphoagronomic and Edaphoclimatic Variables in the Production Systems

The results of the morphoagronomic variables comparison (Table 2) indicate that the maize crops did not develop evenly in the different management systems. 

The plants with the largest diameters were recorded in the MIIC and TT systems, and those with the highest heights in the MIIC, TT, TD, MIIT and TC systems. The MIIC, TT and TD systems presented the largest maize cobs.

**Figure 7:**
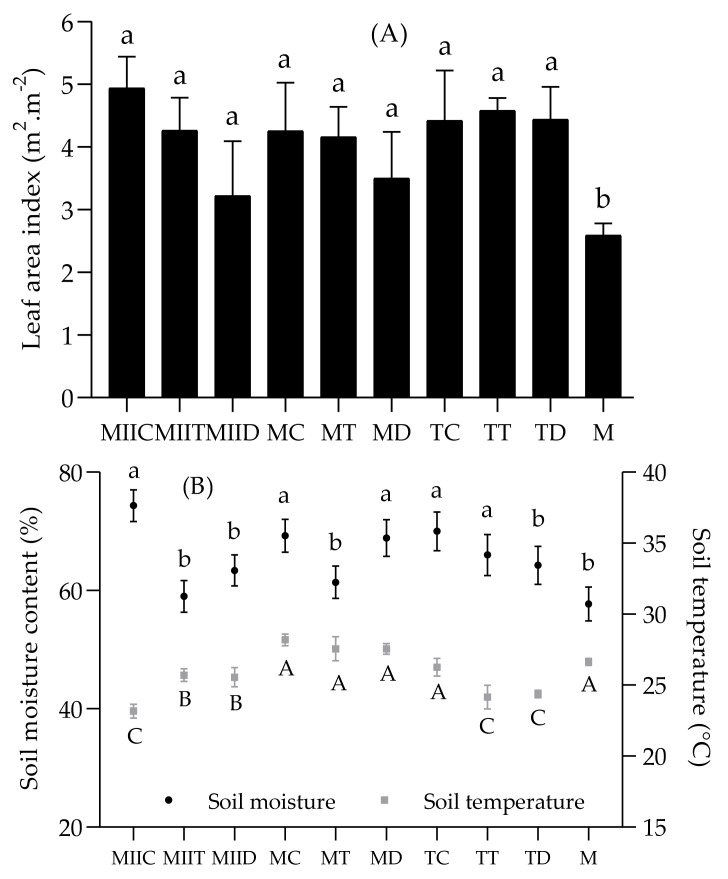
Comparison of Leaf Area Index (**A**) and soil moisture and temperature (**B**) in Push–Pull systems. Different letters indicate a significant difference by the Scott–Knott test (α = 0.05). For abbreviations, see Table 1.

The systems also presented different Leaf Area Index (LAI), soil temperature and moisture (Figure 7). The Push–Pull systems presented similar LAI, which were significantly higher than that of the monoculture (Figure 7A). The highest moisture retentions were recorded in soils of systems with *C*. *juncea* as intercrop (Figure 7B).

### 3.3. Maize and Forage Yields in the Production Systems

**Figure 8:**
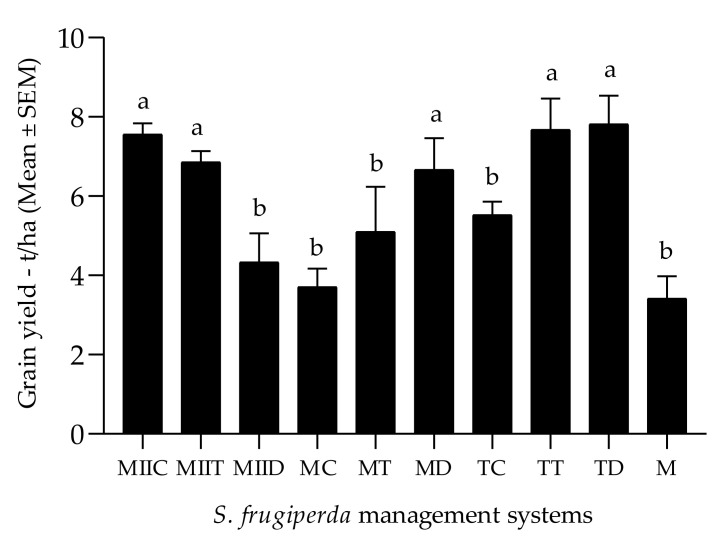
Maize grain yields (Mean ± SEM) in the evaluated management systems. Different letters indicate significant difference (*p* < 0.05) by the Scott–Knott test (α = 0.05). For abbreviations, see Table 1.

Maize grain yields in the systems differed significantly. The lowest yields were observed in the M and MC systems (Figure 8). In relation to the forage grasses, there was a significant difference between their fresh yields. *P*. *maximum* cv. *Mombasa* (54.44 ± 3.77 t/ha) and *P*. *maximum* cv. *Tanzania* (55.56 ± 4.03 t/ha) presented similar yields, which were significantly higher than that of *B*. *hybrid* cv. *Mulato II* (41.11 ± 3.61 t/ha). The only forage species among the intercropped crops (*C*. *juncea*) presented a fresh yield of 5.12 ± 0.13 t/ha.

**Figure 9:**
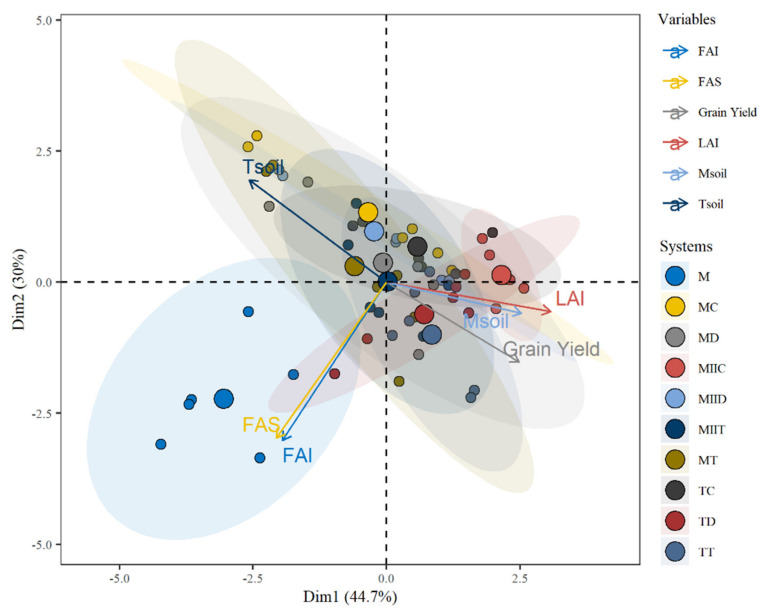
Biplot of principal component analysis based on variables studied in maize Push–Pull systems established in Yautepec, Morelos, Mexico. FAI = Fall Armyworm Incidence; FAS = Fall Armyworm Severity; LAI = Leaf Area Index; Msoil = Soil moisture; Tsoil = Soil temperature. For abbreviations, see Table 1.

The Kaiser criterion suggested the extraction of two main components. These two PCs explain 74.69% of the total variance of the variables. The first PC presented an eigenvalue of 1.64, responsible for 44.65% of the variance; the second PC presented an eigenvalue of 1.34 and explains about 30.04% of the total variance (Figure S2). Based on these two PCs, separations between the management systems were obtained (Figure 9). The relationship between monoculture performance with those of the Push–Pull systems for each of the aspects evaluated is summarized in Table 3.

## 4. Discussion

In this study, the field effectiveness of the Push–Pull systems proposed by Guera et al. [16] for *S*. *frugiperda* management in maize crops in the state of Morelos, Mexico, was evaluated. Push–Pull systems presented lower incidence (Figure 4) and severity (Figure 5 and Figure 6) of fall armyworm, compared to monocultures. The greatest damage reductions were recorded in the MIIC system, which presented a reduction of 69.56% in FAW incidence and a reduction of 58.61% in its severity. These results concur with those of several field effectiveness studies (against Fall Armyworm) of the successful Push–Pull system designed by Khan et al. [12]. Midega et al. [13] and Khan et al. [14] reported reductions of 82.7% in the average number of larvae per plant and 86.7% in the damage of plants per plot in the Push–Pull plots made up of *Brachiaria* cv *Mulato II* and *Desmodium intortum* (Mill.) Urb. Recently, Njeru et al. [39] reported a reduction of more than 50% of fall armyworm infestation in the same Push–Pull system. In the current study, the greatest reductions in Fall Armyworm incidence and severity were recorded in the MIIC, TC, MIID, MC, MIIT and MD systems (Table 3). The low incidence/severity values in all systems with the legume *C*. *juncea* (MIIC, TC, MC) coincide with the results of several studies. Among these are those of Hailu et al. [15], who reported a significant reduction in FAW incidence/severity in maize–legume inter-cropping. Altieri [40] also reported a significant reduction in FAW incidence in maize/beans intercropping.

The greater infestation of monocultures is reflected in the development of their crops. The results of morphoagronomic variables analysis indicates that the maize crops of the Push–Pull systems developed better than the monocultures (Table 2). The lower development of the latter and the existence of intercropping explain why the Push–Pull systems presented significantly higher LAIs (Figure 7A). 

Most Push–Pull systems also outperformed monocultures in terms of maize grain yield (Figure 8), in the ratios of Table 3. This concurs with reports from Africa that Push–Pull (*Pennisetum purpureum* or *Brachiaria* cv *Mulato II* and *Desmodium uncinatum*) yields are 2 times higher than those of monocultures [13,41,42,43]. Improved yields in the Push–Pull systems are due to the reduction in the pest damage and moisture conservation of soil, among others [13]. The MC system maize crops showed poor vegetative development and low average yield, despite low levels of fall armyworm damage and optimal soil temperature and moisture levels. The average yield of this system was affected by its low yield in block III (2.05 t/ha). In that block (the least productive of the three), a high competition between maize and *C*. *juncea* was observed despite periodic pruning applied to *C*. *juncea*. The latter developed better than maize under these adverse conditions. For this reason, it is necessary that studies of optimal densities of *C*. *juncea* be carried out so that maize yield is not affected by the adoption of this system.

Most of the systems with the highest LAI presented high moisture content (MIIC, TT, TC, MC). Unlike Push–Pull systems, maize monocultures presented high soil temperature values and low soil moisture values (Figure 7B), explained by the greater exposure of their soils due to their smaller foliar surface. This concurs with Khan et al. [44], who cite soil moisture conservation and soil temperature reduction among the benefits of Push–Pull systems in Africa. This conservation of soil moisture is essential for the sustainability of agroecosystems [2].

*C*. *juncea*, the only legume of the intercropped species, in addition to fixing nitrogen, serves as green manure. For this reason, the leaves obtained from the periodic pruning applied to it were left on the floor of the plots for soil moisture conservation in the short term and their gradual incorporation into the soil as green manure in the medium or long term.

In the PCA biplot, the Push–Pull systems overlap, which indicates a certain degree of similarity between them. A separation of the monoculture from the Push–Pull systems is also observed (Figure 9). This is because, contrary to the Push–Pull systems, it presented a higher incidence/severity of fall armyworm, higher soil temperature, lower soil moisture content and lower maize grain yield.

The establishment costs of the Push–Pull systems were higher than those of monocultures. However, these costs were offset by the aggregate value of the companion crops. The NPV of all systems was positive and their benefit/cost ratio (BCR) greater than one. This indicates that these systems are economically viable, the most profitable being the systems TD, TT, MD, MIIT and MIIC. These systems, in addition to recovering the investment as in all systems, generated a minimum profit of 70 cents per dollar invested (Table 3). It is also perceptible that these systems, except for MIIC, were the ones that, in addition to presenting high maize yields, generated additional income from the accompanying crops, mainly *T*. *erecta* and *D*. *ambrosioides*, which have higher aggregate values than *C*. *juncea*. The MC system was one of the least profitable and this is because, in addition to presenting a lower maize yield, as previously discussed, it has a companion crop with lower aggregate value. A significant reduction in the costs of establishing the Push–Pull systems is expected in subsequent production cycles, like those reported by Khan et al. [45]. This will make these systems more attractive to maize producers and could favor their mass adoption.

## 5. Conclusions

In most of the Push–Pull systems evaluated, the maize crops developed adequately and those with the highest vegetative cover (LAI) presented a greater soil moisture conservation.

*S*. *frugiperda* incidence and severity in the Push–Pull systems were significantly lower than those observed in monocultures, with *Mulato II*—*C*. *juncea*, *Mulato II*—*D*. *ambrosioides*, *Tanzania*—*C*. *juncea* and *Mombasa*—*C*. *juncea* the ones that best controlled the pest.

The highest maize grain yields and profits were obtained in the *Mombasa*—*D*. *ambrosioides*, *Mulato II*—*T*. *erecta*, *Mulato II*—*C*. *juncea*, *Tanzania*—*T*. *erecta* and *Tanzania*—*D*. *ambrosioides* systems.

## Figures and Tables

**Table 1:** Fall armyworm management systems tested in maize crops in Yautepec, Morelos, Mexico.

System Codes	Components		Treatments
	Attractants ^¥^	Intercrops *	
MIIC	*Brachiaria hybrid* cv *Mulato II*	*Crotalaria juncea*	*Mulato II* & *C*. *juncea*
MIIT		*Tagetes erecta*	*Mulato II* & *T*. *erecta*
MIID		*Dysphania ambrosioides*	*Mulato II* & *D*. *ambrosioides*
MC	*Panicum maximum* cv *Mombasa*	*Crotalaria juncea*	*Mombasa* & *C*. *juncea*
MT		*Tagetes erecta*	*Mombasa* & *T*. *erecta*
MD		*Dysphania ambrosioides*	*Mombasa* & *D*. *ambrosioides*
TC	*Panicum maximum* cv *Tanzania*	*Crotalaria juncea*	*Tanzania* & *C*. *juncea*
TT		*Tagetes erecta*	*Tanzania* & *T*. *erecta*
TD		*Dysphania ambrosioides*	*Tanzania* & *D*. *ambrosioides*
M	*-*	*-*	Maize monoculture not treated with pesticides

Source: Guera et al. [16]; ^¥^ trap plants established at a distance around the main crop; * plants intercropped in the main crop.

**Table 2:** Comparison of morphological variables in the evaluated systems.

Systems	Stem Diameter (cm)	Total Height (m)	Cob Diameter (cm)	Cob Length (cm)
MIIC	2.43 ± 0.11 a	2.54 ± 0.04 a	5.40 ± 0.21 a	24.22 ± 0.77 a
MIIT	2.12 ± 0.09 b	2.43 ± 0.03 a	5.03 ± 0.17 a	22.65 ± 0.40 b
MIID	1.88 ± 0.12 b	2.28 ± 0.06 b	4.94 ± 0.20 b	22.50 ± 0.49 b
MC	2.09 ± 0.11 b	2.18 ± 0.06 b	4.65 ± 0.18 b	22.62 ± 0.36 b
MT	2.07 ± 0.16 b	2.22 ± 0.07 b	4.83 ± 0.17 b	22.43 ± 0.49 b
MD	2.06 ± 0.11 b	2.34 ± 0.04 b	5.28 ± 0.18 a	23.22 ± 0.43 a
TC	2.11 ± 0.13 b	2.45 ± 0.03 a	5.13 ± 0.12 a	22.60 ± 0.37 b
TT	2.42 ± 0.17 a	2.40 ± 0.07 a	5.35 ± 0.21 a	23.39 ± 0.44 a
TD	2.12 ± 0.09 b	2.53 ± 0.04 a	5.43 ± 0.12 a	23.72 ± 0.50 a
M	1.69 ± 0.19 b	2.34 ± 0.05 b	4.51 ± 0.23 b	21.60 ± 0.60 b

Means with different letters in a column indicate significant differences by Scott–Knott test (α = 0.05). For abbreviations, see Table 1.

**Table 3:** Comparison of the performances of Push–Pull systems and maize monocultures.

System	Phytosanitary, Edaphoclimatic and Yield Criteria				Profitability Criteria	
	FAIR (%)	FASR (%)	ASMR (%)	PPY/MY Ratio	NPV ($ USD)	Benefit/Cost Ratio
MIIC	69.56	58.61	28.81	2.21	1012.924	2.62
MIIT	50.00	47.05	2.20	2.00	1065.394	1.90
MIID	61.97	55.56	9.82	1.27	727.265	1.41
MC	60.87	54.88	19.99	1.08	266.900	1.45
MT	46.74	44.23	6.36	1.49	745.613	1.65
MD	52.17	47.50	19.30	1.95	1238.939	1.71
TC	61.96	52.72	21.25	1.61	623.801	2.02
TT	42.39	40.95	14.33	2.24	1250.677	2.06
TD	47.83	44.45	11.33	2.28	1452.002	1.82
M	-	-	-	-	518.703	1.59

FAIR = Fall Armyworm Incidence Reduction; FASR = Fall Armyworm Severity Reduction; ASMR = Additional Soil Moisture Retention; PPY = Push-Pull Yield; MY = Monoculture Yield; NPV = Net Present Value; The conversion of dollar to Mexican peso used was: $1 USD = 18.7018 pesos (December 2019). For abbreviations, see Table 1.

## Data Availability

Data sharing is not applicable to this article.

## Supplementary Materials

The following are available online at https://www.mdpi.com/article/10.3390/insects12040298/s1, Table S1: Data of the establishment of the field experiment in the 2019 maize season (Experimental period: June–December 2019). Table S2. Costs and incomes considered for the profitability analysis of the production systems. Figure S1. Five-point sampling method used in the maize plots (10 × 10 m) of the different production systems. SP = Sampling Point. Figure S2. Scree plots of eigenvalues (A) and cumulative variance explained (B) of principal components of Push–Pull systems evaluation variables at Yautepec, Morelos, Mexico.
