# Supplementary material for: Effectiveness of Push–Pull Systems to Fall Armyworm (Spodoptera frugiperda) Management in Maize Crops in Morelos, Mexico
Source: Insects. 2021 Mar 29;12(4):298. doi: 10.3390/insects12040298 (PMC8067049; doi:10.3390/insects12040298)
Supplement: Supplementary file 1 [file insects-12-00298-s001.pdf]

insects

MDPI

Article

Effectiveness of Push–Pull Systems to Fall Armyworm (*Spodoptera frugiperda*) Management in Maize Crops in Morelos, Mexico

Table S1. Data of the establishment of the field experiment in the 2019 maize season (Experimental period: June - December 2019)

Crop type	Species	Sowing / transplant dates	Distance between plants (cm)	Density (plants/ha)
Trap plants	*B. hybrid* cv *Mulato II*	June 4-8	SS	-
	P. maximum cv. *Mombasa*		SS	-
	P. maximum cv. *Tanzania*		SS	-
Intercropped plants	*T. erecta* §	June 17	80	7 200
	D. ambrosioides §	June 21	40	1350
	C. juncea	June 21 and 22	60	38 400
Main crop	*Z. mays**‡	June 21 and 22	20	60 000

§ Transplanted species;

*Pioneer P3966W;

‡The maize crops of all treatments received foliar fertilization with worm leachate (Dose: 250 liters/ha) on July 10 and 17 and nitrogen fertilization (Dose: 200 kg/ha of Urea corresponding to 92 kg/ha of Nitrogen) on August 14.

SS: sowing in streamlines with a distance of 50 cm between the two sowing lines;

Harvest date: December 16;

Shelling and weighing date: December 18.

Table S2. Costs and incomes considered for the profitability analysis of the production systems

Activities	Values ($USD)
Land preparation ($USD/ha)	136.405
Maize seeds ($USD / ha PP) ¥	48.716
Labor for maize planting ($USD / ha PP) ¥	27.281
Nitrogenous fertilizer ($USD / ha PP) ¥	13.641
Foliar fertilizer ($USD / ha PP) ¥	27.281
Weeding ($USD / ha PP) ¥	115.991
T. erecta cost ($USD / ha PP) ¥	589.268
D. ambrosioides cost ($USD / ha PP) ¥	1178.540
C. juncea seed cost ($USD / ha PP) ¥	27.838
Mombasa seed cost ($USD / ha PP) ª	109.124
Tanzania seed cost ($USD / ha PP) ª	109.124
Mulato II seed cost ($USD / ha PP) ª	109.730
Labor for harvest §	147.579 ($USD / ha) + 6.416 (($USD /ton) × Yield (ton/ha)
Maize price ($USD /ton)	Organic/Agroecological production = 427.766<br>Conventional Production = 294.089
D. ambrosioides income ($USD / ha PP)	1571.386
T. erecta income ($USD / ha PP)	785.691
C. juncea income ($USD / ha PP)	6.991
Income from Mulato II (two cuts) ($USD / ha PP)	57.532
Income from Mombasa/Tanzania (two cuts) ($USD/ha PP)	78.811
Discount rate *	9.5%

¥ Estimated values for the main areas of Push-Pull plots (taken to one hectare); ª Estimated values for the pasture areas of Push-Pull plots (taken to one hectare); §Unisem; * González-Estrada (1999); The conversion of dollar to Mexican peso used was: $ 1 USD = 18.7018 pesos (December 2019).

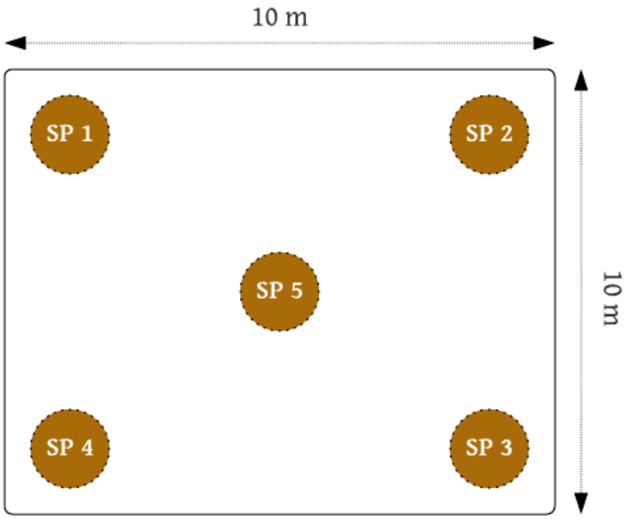

Figure S1. Five-point sampling method used in the maize plots (10 x 10 m) of the different production systems. SP = Sampling Point.

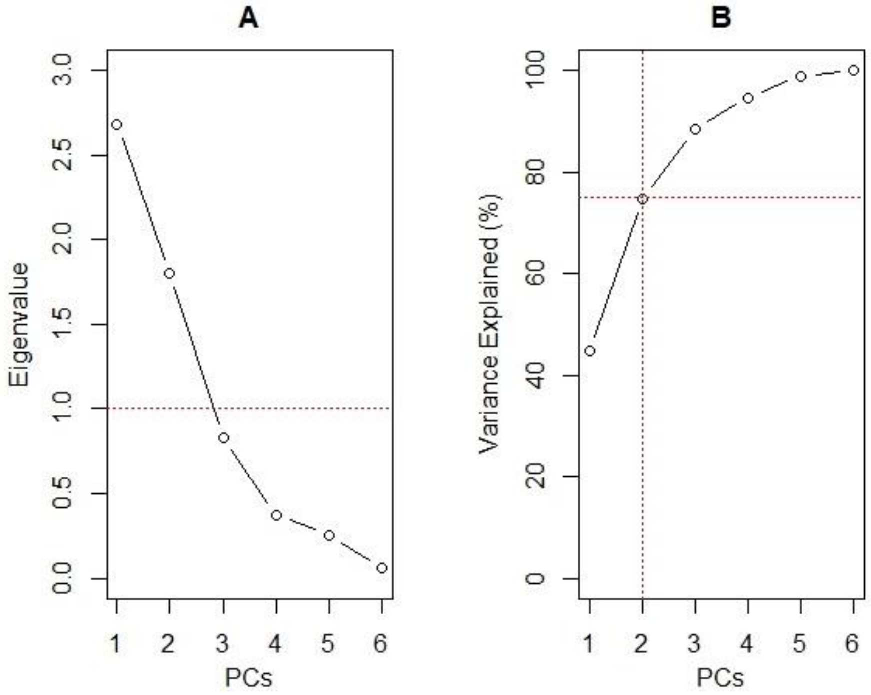

Figure S2. Scree plots of eigenvalues (A) and cumulative variance explained (B) of principal components of Push-Pull systems evaluation variables at Yautepec, Morelos, Mexico.
